# Favorable Response of Metastatic Hepatocellular Carcinoma to Treatment with Trans-arterial Radioembolization Followed by Sorafenib and Nivolumab

**DOI:** 10.7759/cureus.4083

**Published:** 2019-02-16

**Authors:** Charles S Adcock, Louis V Puneky, Garth S Campbell

**Affiliations:** 1 Radiology, University of Mississippi Medical Center, Jackson, USA; 2 Oncology, University of Mississippi Medical Center, Jackson, USA

**Keywords:** hepatocellular carcinoma, y90 transarterial radioembolization, nivolumab, immunotherapy

## Abstract

Trans-arterial radioembolization (TARE) with Y-90 microspheres is an endovascular, liver-directed therapy suitable for treatment of locally advanced hepatocellular carcinoma (HCC) often as a way to reduce tumor size and bridge patients to resection or liver transplant. Opdivo®, or nivolumab, a programmed cell death protein 1 (PD-1) inhibitor, is an immunotherapeutic drug approved in September 2017 for the treatment of HCC in patients who have received prior sorafenib. We report on a patient with hepatocellular carcinoma with right and left portal vein involvement, bony metastasis, and possible lung metastasis. The patient showed a significant response following consecutive treatment with TARE, sorafenib, and nivolumab. Our case suggests that TARE, sorafenib, and nivolumab may have a synergistic effect on the immune response to HCC.

## Introduction

Treatment for hepatocellular carcinoma (HCC) is guided by the Barcelona-Clinic Liver Cancer system, which recommends certain therapies based on the stage of the cancer. Surgical resection and transplant produce the best outcomes but are only recommended for earlier stages of HCC. Other therapies like trans-arterial chemoembolization (TACE) and trans-arterial radioembolization (TARE) can be used to treat HCC confined to the liver or downsize tumors for surgical intervention. Sorafenib, an inhibitor of multiple kinases, is recommended for advanced staged HCC [1]. As of September 2017, the Food and Drug Administration approved nivolumab, an immune checkpoint inhibitor, for patients already taking sorafenib. In this report, we describe the case of a patient with metastatic HCC that showed a favorable response following treatment with TARE, sorafenib, and nivolumab.

## Case presentation

**Figure 1:**
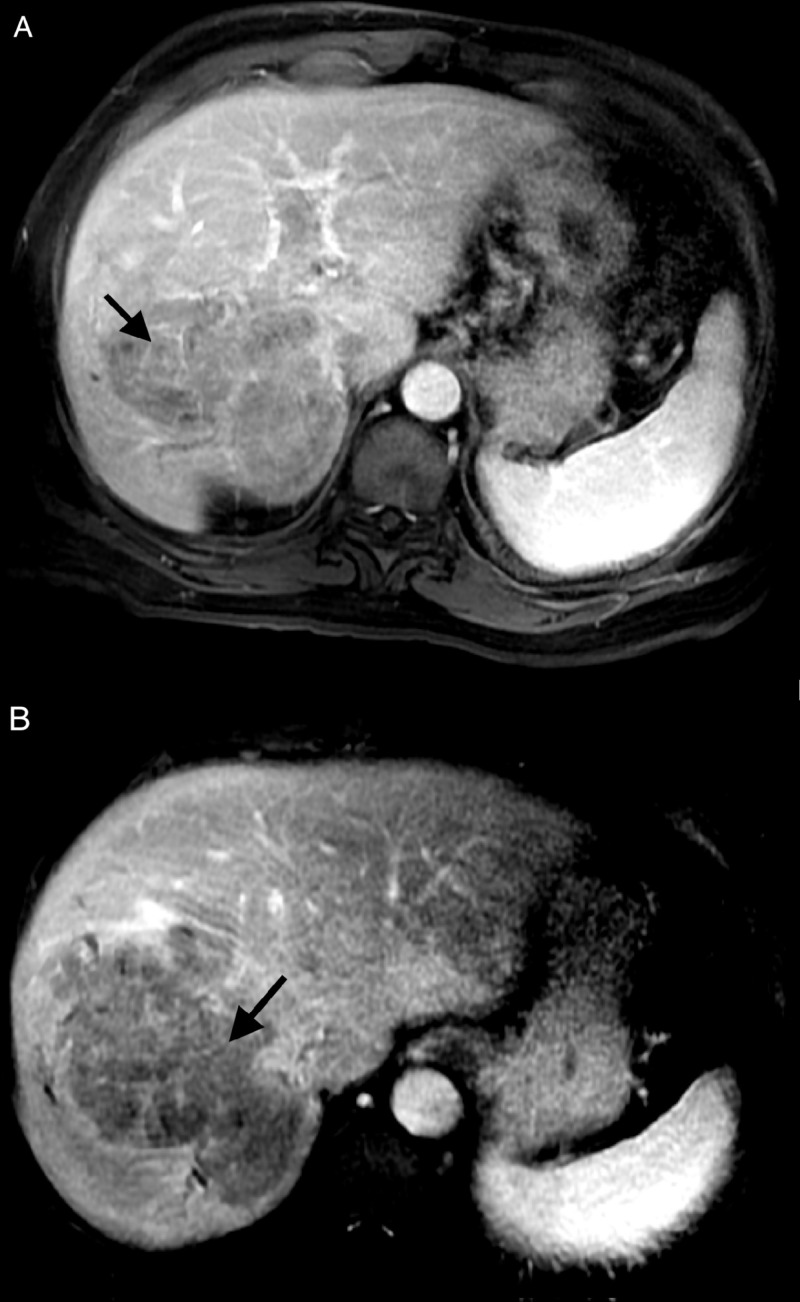
June 2017 MRI with IV contrast A) Arterial phase 20-second LAVA sequence; B) venous phase one-minute LAVA sequence MRI: magnetic resonance imaging; IV: intravenous; LAVA: liver acquisition with volume acquisition

The patient presented in June 2017 with a presumptive diagnosis of HCC based on imaging. A magnetic resonance imaging (MRI) scan of the abdomen revealed a mass greater than 10 cm in size in the right hepatic lobe. The lesion demonstrated arterial phase enhancement (Figure 1A) and venous phase washout (Figure 1B) on contrast imaging characteristic of HCC. Satellite lesions were also noted in the periphery of the right lobe along with tumor thrombus involving both the right and left portal veins. A liver biopsy was not performed. The patient had no history of the liver disease but does have a history of type 2 diabetes mellitus.

**Figure 2:**
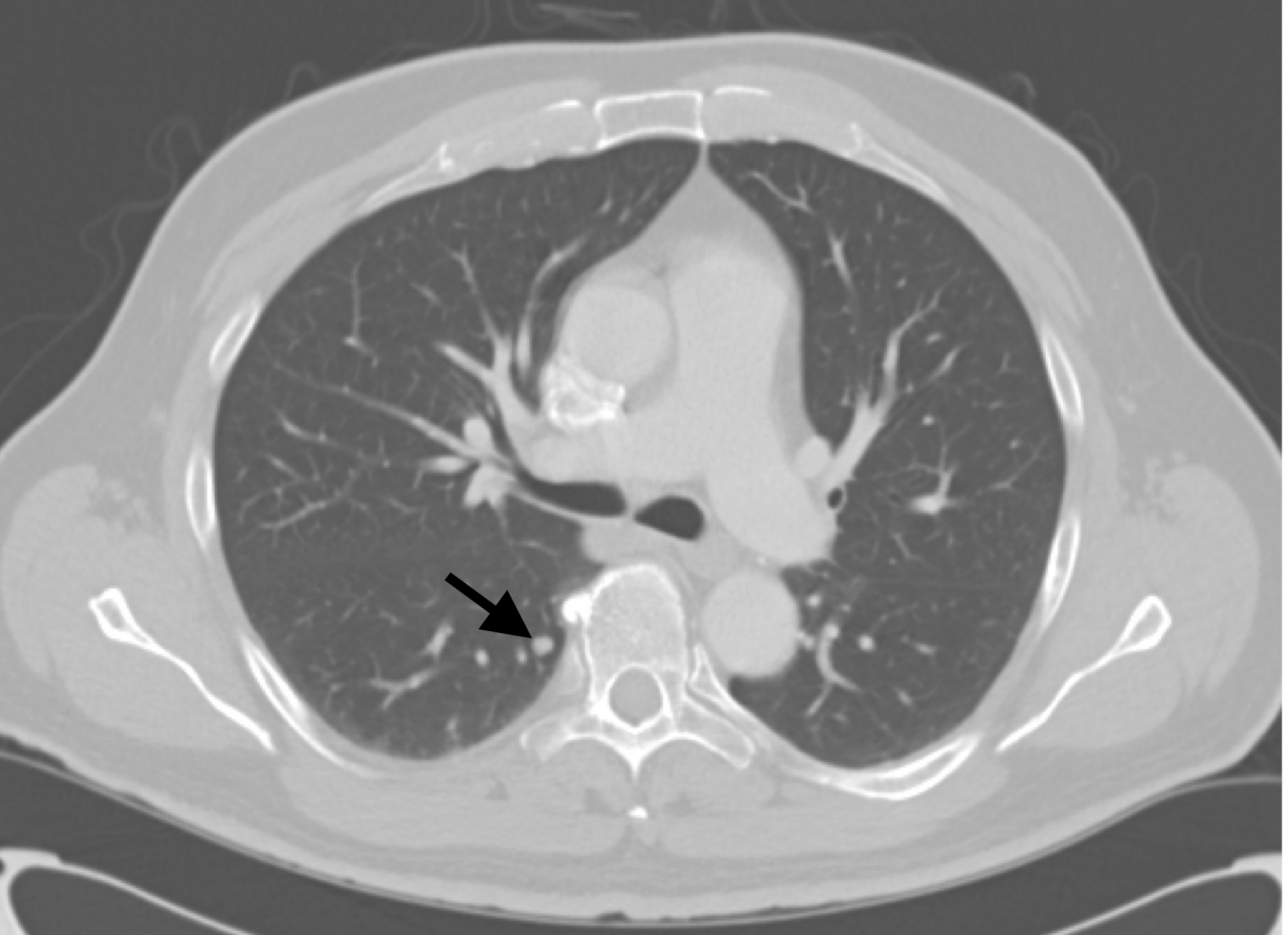
July 2017 CT chest with IV contrast showing a 6-mm lung nodule (arrow) CT: computed tomography; IV: intravenous

**Figure 3:**
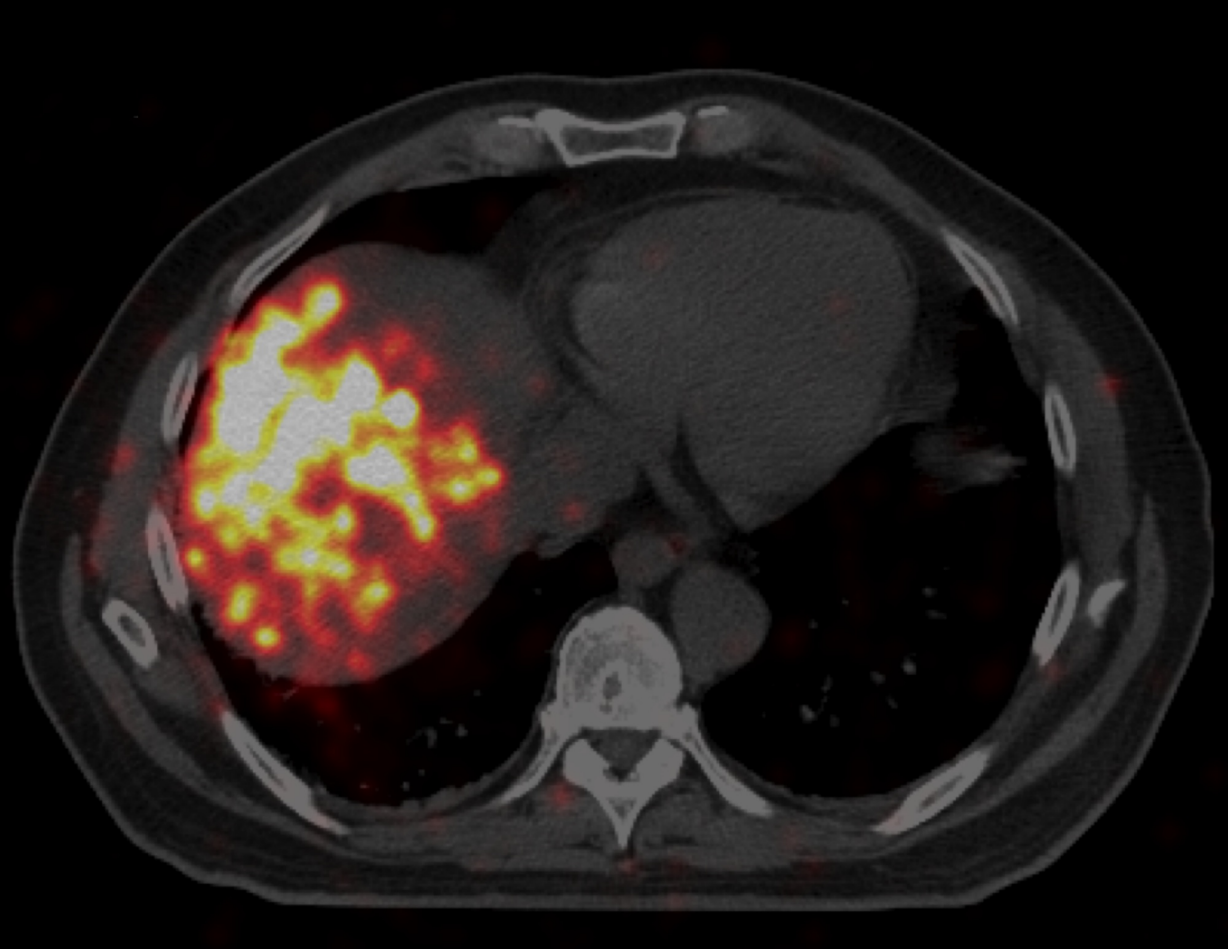
July 2017 PET/CT (scan of Y-90 activity) two hours post Y-90 PET: positron emission tomography, CT: computed tomography

In July, a chest CT scan was performed that showed multiple pulmonary nodules too small for biopsy, the largest up to 6-mm diameter (Figure 2). The patient was informed that these could be metastases and still decided to proceed with a transarterial radioembolization (TARE) treatment. The patient was scheduled for TARE with glass Y-90 microspheres and underwent planning arteriogram for dosimetry, estimation of lung shunt, and prophylactic, coil embolization of gastroduodenal and right gastric arteries. The dose prescribed was 100 Gy to the right hepatic lobe. TARE was performed successfully delivering 93 Gy to the right hepatic lobe with an administered activity of 84.9 mCi. A positron emission tomography (PET) scan performed two hours after the TARE procedure showed Y90 activity in the targeted right hepatic lobe (Figure 3).

**Figure 4:**
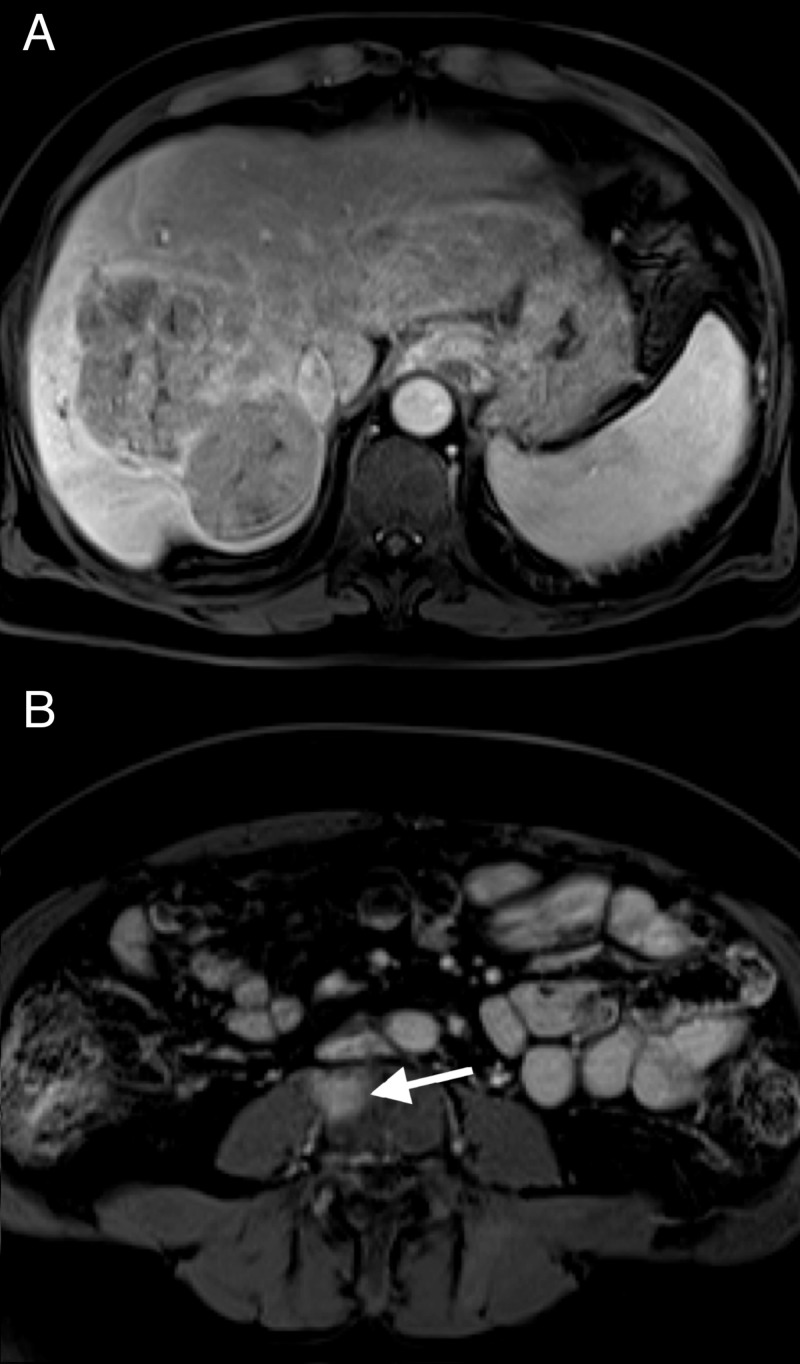
September 2017 MRI with IV contrast arterial phase 20-second (T1) vibe sequence (A) Reduced vascularity of right hepatic lobe tumor; (B) new L4 lesion MRI: magnetic resonance imaging, IV: intravenous

In September, the patient’s alpha-fetoprotein (AFP) was elevated to >18,000 ng/mL. In late September, about 2.5 months post-TARE, the patient received another MRI of the abdomen, this time showing a reduction in vascularity of the primary right hepatic lobe lesion (Figure 4A). This scan also revealed a lesion in the L4 vertebral body compatible with metastasis (Figure 4B).

**Figure 5:**
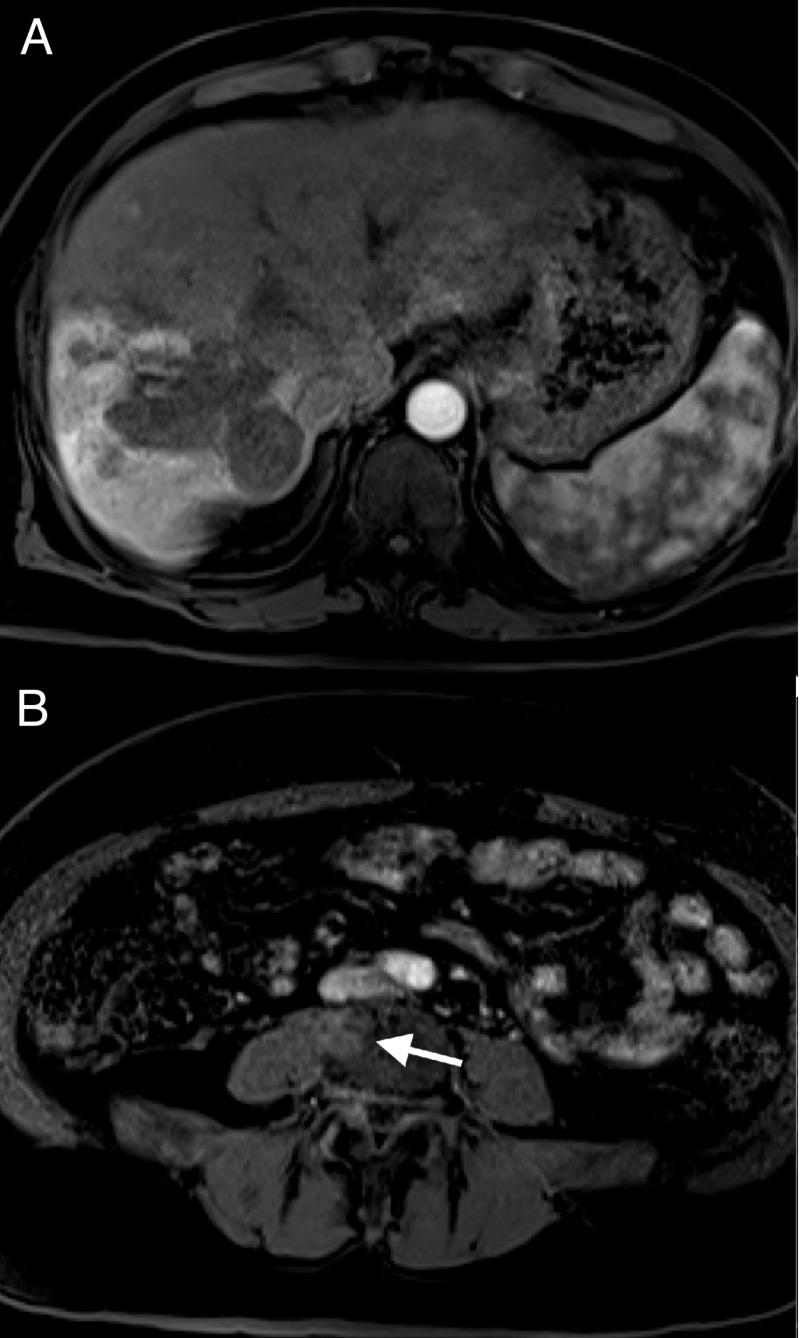
December 2017 MRI with IV contrast arterial phase 20 second (T1) vibe sequence (A) Contraction of the right hepatic lobe with perfusional changes from Y-90, decreased right hepatic lobe tumor size, and markedly reduced enhancement; (B) decreased enhancement of the L4 lesion MRI: magnetic resonance imaging, IV: intravenous

In October, the patient’s AFP was >25,000 ng/mL. Enlargement of the lung nodules was seen on chest CT, the largest being 8.8 mm. The patient was prescribed two 200 mg tablets of sorafenib by mouth daily for one month starting in October and ending in November. The patient transferred care to another hospital in November and was switched to biweekly infusions of nivolumab at 240 mg/24 mL. In late December, three months after the previous MRI, another MRI of the abdomen showed that the primary liver lesion had lost all vascular enhancement (Figure 5A), and the vertebral metastasis to L4 was stable (Figure 5B).

**Figure 6:**
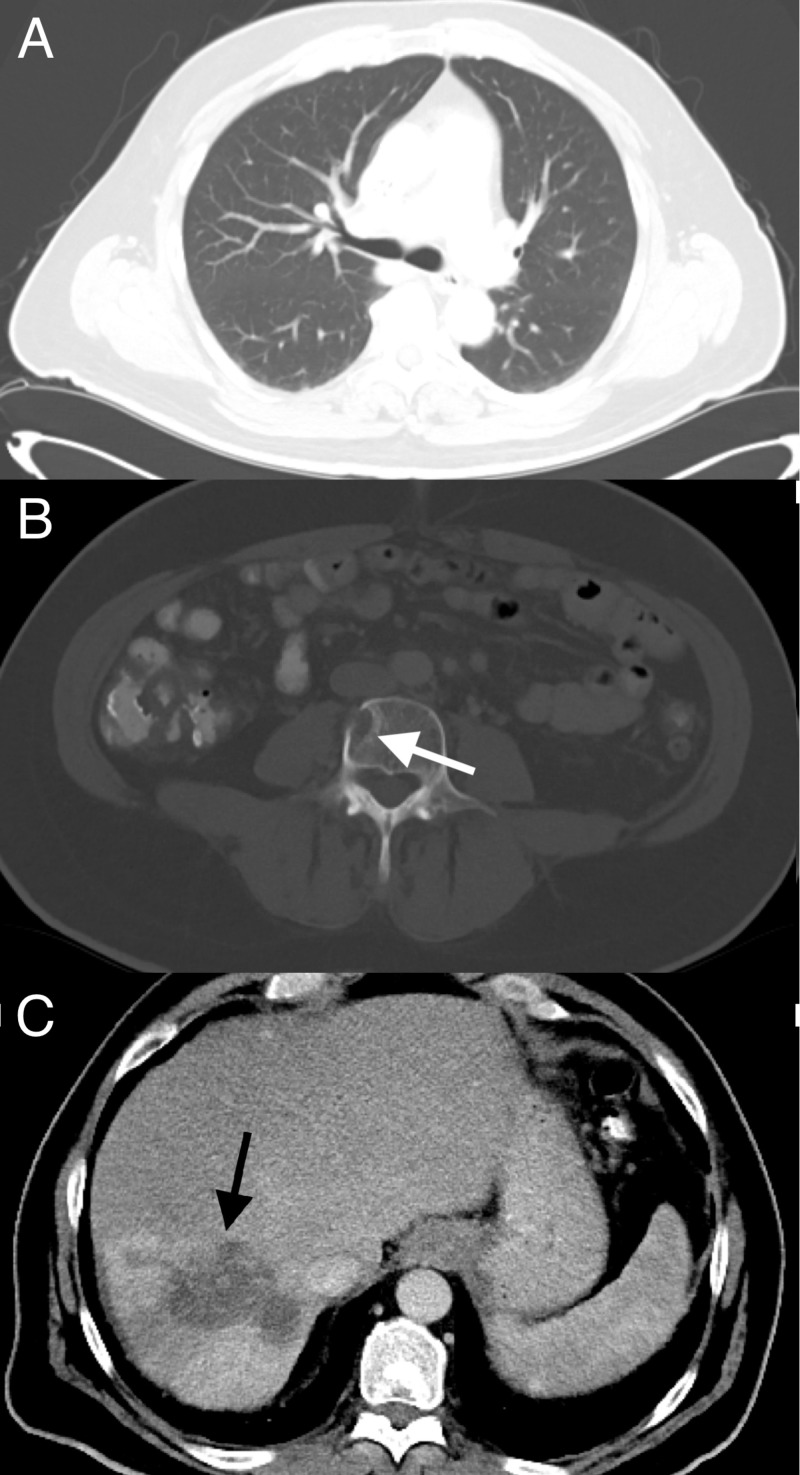
February 2018 CT CAP with IV contrast (A) Disappearance of the 6-mm lung lesion; (B) persistent lytic L4 lesion; (C) further decrease in size of the right hepatic lobe and right hepatic lobe lesion CT: computed tomography, CAP: chest, abdomen, and pelvis, IV: intravenous

**Figure 7:**
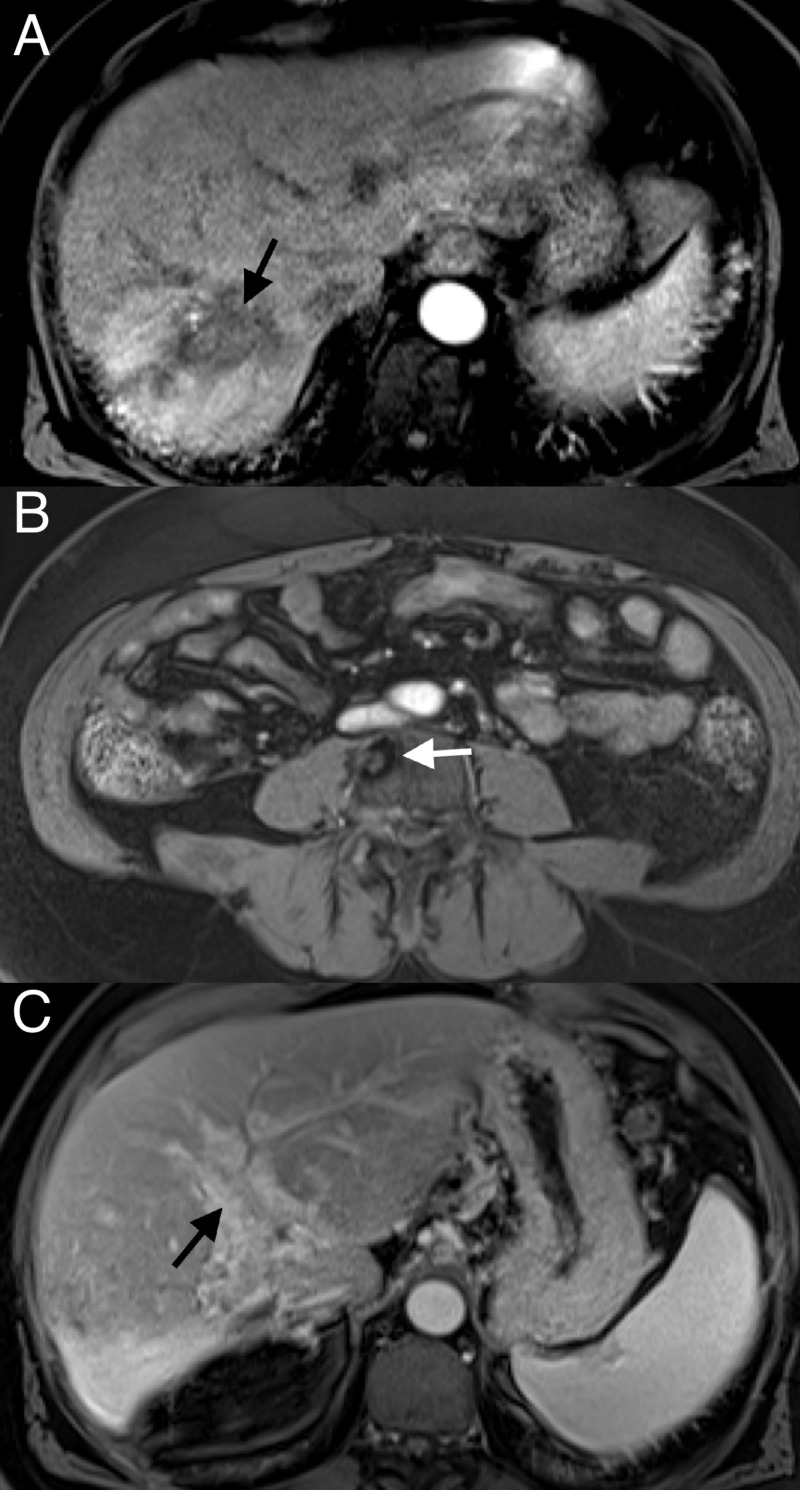
June 2018 MRI with IV contrast arterial phase 20 second (T1) vibe sequence (A) Further decrease in tumor size; (B) decreased enhancement of L4 lesion; (C) venous phase 70-second (T1) vibe sequence showing cavernous transformation of the portal vein and no clear evidence of residual tumor thrombus (chronic occlusion) MRI: magnetic resonance imaging, IV: intravenous

In February, the patient received a CT scan, notably showing an absence of the previously noted lung nodules (Figure 6A). The AFP level in April 2018 was markedly reduced to 2.1 ng/mL. An MRI of the abdomen in June showed an overall favorable response to treatment with a decrease in size in the right hepatic lobe mass (Figure 7A). The L4 lesion was noted to have remained unchanged (Figure 7B), and the right and left portal vein thrombosis were noted to have remained unchanged (Figure 7C). On CT, the L4 lesion had undergone slight sclerotic changes. The patient has chronic portal vein occlusion with cavernous transformation, but there is no clear evidence of tumor thrombus. The AFP in June was 2.0 ng/mL.

The most recent MRI in September showed similar findings to the one in June with a stable L4 lesion. The AFP in September was 2.3 ng/mL. The drop in AFP from a high of >25,000 to 2.3 ng/mL along with the disappearance of lung nodules, reduction in primary tumor size, and lack of progression of the L4 lesion all suggest a favorable and sustained response to treatment.

## Discussion

Nivolumab is a programmed cell death protein 1 (PD-1) inhibitor. Nivolumab’s inhibition of PD-1 is thought to be the reason behind its ability to increase T-cell response against various cancers. Treatment response to immunotherapy, like with nivolumab, is often delayed and can show increased overall survival, while not showing much improvement in progression-free survival. Gulley et al. suggest that this slow response may be due to a phenomenon, known as antigen spreading. Antigen spreading refers to the immune system’s continual identification of additional cancer-specific antigens as several cancer cells are killed and identified [2].

The process by which cancer cells die and present antigen that generates an immune response is referred to as immunogenic cell death (ICD) [3]. Chemotherapy and radiation have been shown to enhance ICD [4-5]. In the presence of therapeutic doses of radiation, some cancer cells undergo changes that signal dendritic cells to kill and phagocytose the cancer cells. Thus, these radiation-induced changes lead to increased antigen presentation of cancer-specific antigen to T-and B-cells [3,6]. Golden et al. showed in an in vitro study that immunogenic cell death was radiation dose-dependent and enhanced chemotherapy-induced ICD [7].

A case report published in May 2018 by Wehrenberg-Klee et al. describes a case of advanced hepatocellular carcinoma that was successfully bridged to partial hepatectomy with a combination of nivolumab and TARE with Y90. They confirmed a complete response with surgical pathology showing negative margins. They hypothesized that the combination therapy may enhance the immune response to HCC [8].

## Conclusions

The prior transarterial radioembolization (TARE) and sorafenib could have created a favorable environment for immunogenic cell death (ICD). A large number of previous ICDs could have expedited the usually slow process of antigen spreading that has been associated with immunotherapeutic drugs like nivolumab. It is possible that a synergistic relationship exists between TARE, sorafenib, and nivolumab, and it could be responsible for the robust treatment response to HCC seen in this patient.
